# Laboratory virulence of entomopathogenic nematodes to the sweetpotato whitefly, *Bemisia tabaci*

**DOI:** 10.21307/jofnem-2021-096

**Published:** 2021-11-26

**Authors:** Yinping Li, George N. Mbata, David I. Shapiro-Ilan

**Affiliations:** 1 Agricultural Research Station, Fort Valley State University, 1005 State University Drive, Fort Valley, GA 31030; 2 USDA, Agricultural Research Service, Southeastern Fruit and Tree Nut Research Laboratory, 21 Dunbar Road, Byron, GA 31008

**Keywords:** *Bemisia tabaci*, Biological control, Entomopathogenic nematode, Laboratory virulence, Plant protection, Snap bean, Sweetpotato whitefly, Tomato

## Abstract

The sweetpotato whitefly, *Bemisia tabaci* Middle East-Asia Minor 1 (MEAM1), is a major insect pest on vegetable crops worldwide. Enormous economic losses result from direct and indirect plant damage caused by MEAM1. Biological control using entomopathogenic nematodes (EPN) may be an effective alternative strategy against MEAM1 because this pest has developed resistance to most insecticides. First, nine EPN species (*Heterorhabditis bacteriophora*, *H. indica*, *H. georgiana*, *H. floridensis*, *Steinernema feltiae*, *S. carpocapsae*, *S. riobrave*, *S. glaseri*, and *S. rarum*) were investigated for virulence to MEAM1 third instar nymphs on snap bean leaves under laboratory conditions. The mortality of MEAM1 nymphs was evaluated at 3 days post-inoculation (dpi). Compared to the water control, the application of the nine EPN species except *S. glaseri* resulted in significantly higher mortality of MEAM1 nymphs, such as *H. bacteriophora* (66.31%), *H. floridensis* (56.38%), *S. carpocapsae* (54.54%), and *S. rarum* (57.80%). Subsequently, the four virulent EPN species, *H. bacteriophora*, *H. floridensis*, *S. carpocapsae*, and *S. rarum* were evaluated further for virulence to MEAM1 nymphs on snap bean and tomato leaves. The mortality of MEAM1 nymphs was assessed at 3 dpi and 7 dpi. There were no significant differences in MEAM1 nymphal mortality between tomato and snap bean at either 3 dpi or 7 dpi. The mortality of MEAM1 nymphs caused by the application of *H. floridensis* (99.25%) was significantly higher than the other three EPN species and the water control at 7dpi. The results indicate that *H. floridensis* is a very promising biocontrol agent for *B. tabaci* management.

The sweetpotato whitefly, *Bemisia tabaci* Gennadius (Hemiptera: Aleyrodidae) Middle East-Asia Minor 1 (MEAM1), is one of the most economically important insect pests of various vegetable crops on a global scale (Byrne and Bellows, 1991; Henneberry and Castle, 2001; Robertson et al., 2014; Li et al., 2021). The insect causes extensive plant damage by direct feeding on plants, secreting honeydew, generating plant disorders, and transmitting plant viruses (Schuster et al., 1996; Walker et al., 2009; Perring et al., 2018). The direct and indirect plant damage results in significant economic losses (Schuster, 1992; Schuster et al., 1996; Henneberry and Faust, 2008; Little, 2019a, 2019b). MEAM1 management relies mainly on chemical insecticides (Palumbo et al., 2003; Perring et al., 2018; Horowitz et al., 2020). However, the pest has developed considerable resistance to several classes of chemical insecticides (Horowitz et al., 2007, 2020; Palumbo et al., 2001). Therefore, alternative control methods are warranted, such as biological control using entomopathogenic nematodes (EPN).

EPN in the genera *Heterorhabditis* and *Steinernema* are important biological control agents that are utilized to manage a variety of devasting insect pests (Shapiro-Ilan et al., 2002; Grewal et al., 2005; Shapiro-Ilan et al., 2020). These nematodes are lethal parasites and kill insect pests with the assistance of mutualistic bacteria (Kaya and Gaugler, 1993). The infective juveniles (IJs), the only free-living stage, enter the host through mouth, anus, spiracles or the insect cuticle (Bedding and Molyneux, 1982). Once inside the host, the bacteria are released, and the insect usually dies within 24–48 h due to toxemia or septicemia (Kaya and Gaugler, 1993). After nutrition in the host is depleted, newly emerged IJs exit the host to find new insects to attack (Kaya and Gaugler, 1993).

EPN have been demonstrated to play a critical role in *B. tabaci* management on vegetables (Cuthbertson et al., 2003a, 2003b; Head et al., 2004; Cuthbertson and Walters, 2005; Cuthbertson et al., 2007a, 2007b, 2008; Qiu et al., 2008; Ruiz-Platt and Cabello, 2009). For example, the application of *S. feltiae* Filipjev (Rhabditida: Steinernematidae) resulted in high mortality of *B. tabaci* nymphs on tomato [*Solanum lycopersicum* L. (Solanales: Solanaceae)] under both controlled laboratory (> 90%) and glasshouse (> 80%) conditions (Cuthbertson et al., 2007b). However, the efficacy of EPN against *B. tabaci* was not consistently high. It was reported that the mortality of *B. tabaci* nymphs in pepper (Solanales: Solanaceae) caused by *S. feltiae* was only 18.48% (Ruiz-Platt and Cabello, 2009).

Because less than five EPN species have been previously evaluated against *B. tabaci* and the results were mixed, there is a need to screen more EPN species to determine those that exhibit consistent high virulence to *B. tabaci*. Therefore, we conducted a broader virulence screening using nine EPN species under laboratory conditions. Moreover, host plant characteristics are known to impact EPN efficacy against *B. tabaci* (Head et al., 2004; Cuthbertson et al., 2007b; Qiu et al., 2008). For instance, the effect of *S. feltiae* on *B. tabaci* was significantly influenced by the host plants based on the leaf surface characteristics (Head et al., 2004; Qiu et al., 2008). Thus, we considered host plant as an impact factor and investigated the virulence of EPN to MEAM1 on the infested leaves of two commercially important vegetables {snap bean [*Phaseolus vulgaris* L. (Fabales: Fabaceae)] and tomato} cultivated in the southern United States.

## Materials and methods

### *Bemisia tabaci* MEAM1 colony

The MEAM1 colony was originally acquired from the USDA-ARS Southeastern Fruit and Tree Nut Research Laboratory culture collection in Byron, GA in August 2020. After that, the colony was maintained in the mesh cages [Dimension: 60 cm × 60 cm × 60 cm (length × width × height), mesh size: 160 µm aperture; Bioquip Products Inc., Compton, CA] on tomato plants under laboratory conditions at 25 ± 2°C with a 16:8 hours light: dark (L: D) regime. The laboratory MEAM1 colony was supplemented with field MEAM1 colony collected from the snap bean field in October 2020 at Fort Valley State University Research Farm off Camp John Hope Road (Fort Valley, GA) since the field populations of *B. tabaci* in the state of Georgia, United States are all exclusively MEAM1 (A. N. Sparks, University of Georgia, pers. Comm., 2020).

### Entomopathogenic nematodes

The nine species used in this study were reared at 25 ± 2°C on last instar of greater wax moth, *Galleria mellonella* L. (Lepidoptera: Pyralidae), according to the established procedures (Woodring and Kaya, 1988). The larvae of *G. mellonella* were obtained from Webster’s Waxie Ranch (Webster, WI). All the nematodes were stored at 10°C for less than 15 days prior to use. The nine species used were: *Heterorhabditis bacteriophora* Poinar (Rhabditida: Heterorhabditidae) (VS strain); *H. indica* Poinar, Karunakar, and David (HOM1 strain); *H. georgiana* Nguyen, Shapiro-Ilan, and Mbata (Kesha strain); *H. floridensis* Nguyen, Gozel, Koppenhöfer, and Adams (K22 strain); *S. feltiae* (SN strain); *S. carpocapsae* Weiser (All strain); *S. riobrave* Cabanillas, Poinar, and Raulston (355 strain); *S. glaseri* Steiner (VS strain); and *S. rarum* (17C&E strain). All the nematodes were obtained from the USDA-ARS Southeastern Fruit and Tree Nut Research Laboratory culture collection in Byron, GA.

### Experiment design and procedures

In the first virulence experiment, the virulence of nine EPN species to MEAM1 third instar nymphs was investigated on snap bean leaves under laboratory conditions. The third instar of MEAM1 nymphs was the target life stage because the second and third instars have been proved to be the most susceptible to EPN infections (Cuthbertson et al., 2007a). The experiment was conducted three times (three trials over time). Each trial involving three replications was arranged as a completely randomized design and one leaf served as a replication. The ten treatments included a water control and nine EPN species described above. The application rate of each EPN species was 10,000 IJs/ml suspended in distilled water.

The MEAM1 infested snap bean leaves (about 20 third nymphs per leaf) were collected from the snap bean plants at Fort Valley State University Research Farm (Fort Valley, GA). Each infested snap bean leaf was sprayed with the treatment suspensions thoroughly until runoff using 946-ml plastic spray bottles (Delta Industries, King of Prussia, PA). Each snap bean leaf was placed into a glass jar [Dimension: 6.5 cm × 17 cm (diameter × height); Mason Jars Company, Erie, PA] filled with water. Each jar was placed into a 7.6-L plastic container (WebstaurantStore, Lititz, PA) and about 30 mL of water was added into the bottom of the 7.6-L plastic container to maintain moisture inside. Thereafter, the lids of the 7.6-L plastic containers with 10 perforations (Diameter: 0.5 mm) for ventilation were replaced as covers for the containers. All the 7.6-L plastic containers were placed in a cooled incubator set at 25 ± 2°C and 16 L: 8 D. The relative humidity in the 7.6-L plastic containers was detected as 97 ± 2% by the temperature and humidity meter HTC-1 (‎Niceeshop; online store). At 3 days post-inoculation (dpi), the nymphs on each leaf were checked to determine whether they were dead under a dissecting microscope (Nikon SMZ1000; BioQuip Products, Inc., Rancho Dominguez, CA). If the nymphs cannot move after probing using a needle, the nymphs were regarded to be dead.

In the second virulence experiment, four EPN species [*H. bacteriophora* (VS strain), *H. floridensis* (K22 strain), *S. carpocapsae* (All strain), and *S. rarum* (17C&E)] with high virulence to MEAM1 nymphs were selected based on the outcome of the first virulence experiment above. The virulence to MEAM1 third instar nymphs was assessed on snap bean and tomato leaves. The first evaluation was conducted three times (3 trials over time) and checked at 3 dpi. Because the mortality of MEAM1 nymphs caused by the EPN at 3  dpi in the first evaluation was relatively low (11.59–30.16%), the second evaluation was conducted and the exposure time to the treatments was extended from 3 days to 7 days. There were 2 trials (over time) in the second evaluation. Each trial with three replications was arranged in a completely randomized design. Each trial consisted of a two-way factorial treatment structure that included all combinations of two host plants (tomato and snap bean) and five treatments [*H. bacteriophora* (VS strain), *H. floridensis* (K22 strain), *S. carpocapsae* (All strain), *S. rarum* (17C&E), and water control]. The application rate of each EPN species was 10,000 IJs/ml suspended in distilled water.

The MEAM1 infested tomato and snap bean leaves (approximately 20 third nymphs per leaf) were excised from the laboratory-grown snap bean and tomato plants. Each infested leaf was sprayed thoroughly until runoff using the treatment suspensions in 946-ml plastic spray bottles. Following the applications of the treatments on the leaves, the petiole of each leaf was inserted into 2% water agar in a Petri dish [Dimension: 10 cm × 2 cm (diameter × height); Pyrex, Greencastle, PA]. Because the short petioles of tomato leaves cannot be inserted into the jars, the experiment method was revised to use 2% water agar in the Petri dishes to keep leaves alive. Each Petri dish with a leaf was transferred into a one-gallon Ziploc bag containing a wet paper towel at the bottom. All the Ziploc bags were sealed and transferred into an incubator with environmental condition as stated above. The relative humidity in the Ziploc bags was detected as 98 ± 2% using the temperature and humidity meter HTC-1. At 3 dpi or 7 dpi, the number of dead MEAM1 nymphs was checked using the method described in the first virulence experiment above.

### Data analysis

In the first virulence experiment, a generalized linear mixed model assuming a binomial distribution was fitted to the response variable defined as the number of dead MEAM1 nymphs from the initial number that exposed to the treatments. A logit link function was used to estimate the mortality of MEAM1 nymphs. The fixed effects in the linear predictor included trial, treatment, and the two-way interaction. The related random effects were included to account for over-dispersion in the data.

In each evaluation (3 dpi and 7 dpi) of the second virulence experiment, the generalized linear mixed model and the logit link function were the same as that in the first virulence experiment above. The fixed effects consisted of trial, host plant, treatment, as well as all two- and three-way interactions. To account for over-dispersion, the related random effects were identified.

In each virulence experiment above, no evidence of over-dispersion was determined following assessment with maximum-likelihood fit statistic Pearson Chi-Square/DF. The final statistical model used for inference was fitted using Residual Pseudo-likelihood. The statistical model was fitted in the PROC GLIMMIX procedure using the SAS 9.4 statistical software (SAS Institute, Cary, NC). Pairwise comparisons were conducted using Tukey-Kramer’s adjustments for main effect comparisons, to avoid inflation of type I error due to multiple comparisons.

## Results

### First virulence experiment

**Figure 1:**
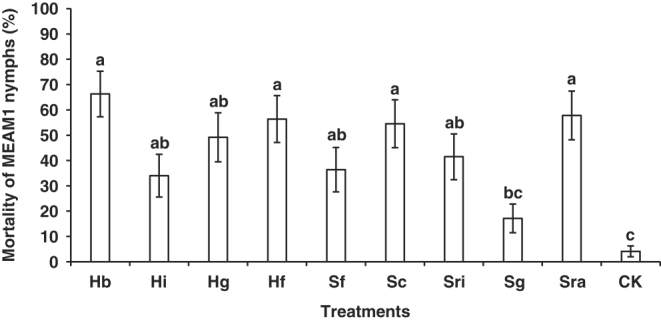
Mortality of *Bemisia tabaci* Middle East-Asia Minor 1 (MEAM1) nymphs (%) on snap bean leaves caused by the ten treatments at 3 days post-inoculation in the first virulence experiment. Treatments: Hb = *Heterorhabditis bacteriophora* (VS strain), Hi = *H. indica* (HOMl strain), Hg = *H. georgiana* (Kesha strain), Hf = *H. floridensis* (K22 strain), Sf = *Steinernema feltiae* (SN strain), Sc = *S. carpocapsae* (All strain), Sri = *S. riobrave* (355 strain), Sg = *S. glaseri* (VS strain), Sra = *S. rarum* (17C&E strain), CK = Water control. Different letters above bars indicate statistical significance (*P* < 0.05) among the treatments.

No significant interaction between trial and treatment was identified (*F* = 1.83; df=18, 51.63; *P* = 0.05). So, the data from the repeated trials were combined. Significant differences were associated with the mortality of MEAM1 nymphs among the ten treatments (*F* = 6.01; df=9, 53.06; *P* < 0.0001). Compared to the water control, the applications of the nine EPN species except *S. glaseri* (VS strain) resulted in significantly higher mortality of MEAM1 nymphs, such as *H. bacteriophora* (VS strain) [Mean (± SEM) (%), 66.31 (± 9.00)%], *H. floridensis* (K22 strain) [56.38 (± 9.23)%], *S. carpocapsae* (All strain) [54.54 (± 9.47)%], and *S. rarum* (17C&E strain) [57.80 (± 9.61)%] (Fig. 1).

### Second virulence experiment

**Figure 2:**
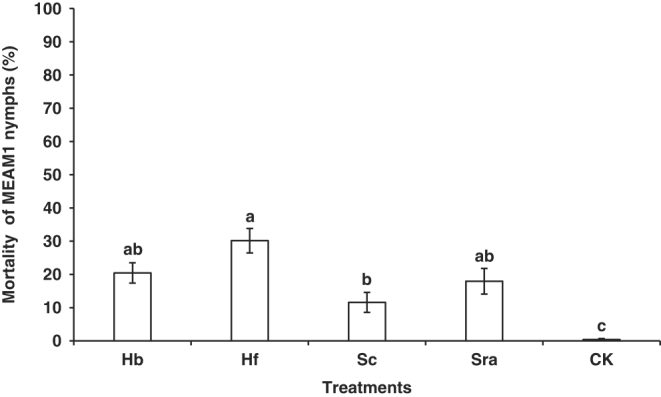
Mortality of *Bemisia tabaci* Middle East-Asia Minor 1 (MEAM1) nymphs (%) caused by the five treatments at 3 days post-inoculation in the second virulence experiment. Treatments: Hb = *Heterorhabditis bacteriophora* (VS strain), Hf = *H. floridensis* (K22 strain), Sc = *Steinernema carpocapsae* (All strain), Sra = *S. rarum* (17C&E strain), CK = Water control. Different letters above bars indicate statistical significance (*P* < 0.05) among the treatments.

At 3 dpi, no significant two-way or three-way interactions were observed among trial, host plant, and treatment (Table 1). Thus, the data from repeated trials were combined and the main effect of treatment was compared across these factors (trial and host plant). The mortality of MEAM1 nymphs was not significantly different between snap bean and tomato (Table 1). However, there were significant differences on the mortality of MEAM1 nymphs among the five treatments (*F* = 3.48; df  =  4, 24; *P* = 0.022) (Fig. 2). Compared with the water control, the applications of the four EPN species resulted in significantly higher mortality of MEAM1 nymphs, such as *H. floridensis* (K22 strain) [30.16 (± 3.67) %] (Fig. 2).

**Table 1.** Three-way Analysis of Variance (ANOVA) table for the second virulence experiment at 3 days post-inoculation (dpi) and 7 dpi.

Effect	3 dpi			7 dpi		
	*F*	df	*P*	*F*	df	*P*
Trial	19.86	2, 6	0.0023	0.00	1, 4	0.99
Treatment	3.48	4, 24	0.022	10.13	4, 36	<0.0001
Host plant	0.00	1, 30	0.99	0.00	1, 36	0.99
Trial × Host plant	0.05	2, 30	0.95	0.00	1, 36	0.99
Trial × Treatment	0.75	8, 24	0.65	2.05	4, 36	0.11
Treatment × Host plant	1.29	4, 30	0.30	2.14	4, 36	0.10
Trial × Host plant × Treatment	1.16	8, 30	0.35	1.61	4, 36	0.19

**Figure 3:**
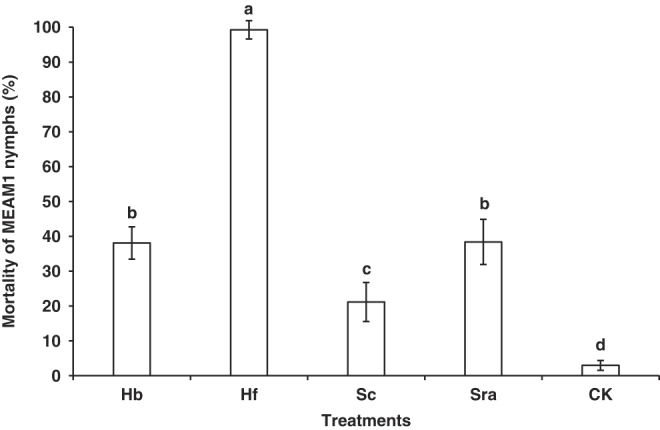
Mortality of *Bemisia tabaci* Middle East-Asia Minor 1 (MEAM1) nymphs (%) caused by the five treatments at 7 days post-inoculation in the second virulence experiment. Treatments: Hb = *Heterorhabditis bacteriophora* (VS strain), Hf = *H. floridensis* (K22 strain), Sc = *Steinernema carpocapsae* (All strain), Sra = *S. rarum* (17C&E strain), CK = Water control. Different letters above bars indicate statistical significance (*P* < 0.05) among the treatments.

At 7 dpi, there were no significant two-way or three-way interactions among trial, host plant, and treatment, nor was there any main effect of host plant (Table 1). Thus, the data from repeated trials were combined and main effect of treatment was compared across these factors (trial and host plant). There were significant differences associated with the mortality of MEAM1 nymphs among the five treatments (*F* = 10.13; df  =  4, 36; *P* < 0.0001) (Fig. 3). The application of *H. floridensis* (K22 strain) caused significantly higher mortality of MEAM1 nymphs [99.25 (± 2.63) %] than the other three EPN species and the water control (Fig. 3).

## Discussion

This laboratory study is the first broad virulence screening of EPN species against MEAM1 on the vegetable leaves, involving nine EPN species, which is a broader screening than that in previous studies (Cuthbertson et al., 2003a, 2003b; Head et al., 2004; Cuthbertson and Walters, 2005; Cuthbertson et al., 2007a, 2007b, 2008; Qiu et al., 2008; Ruiz-Platt and Cabello, 2009). Moreover, this is the first report of evaluating the virulence of *H. floridensis* (K22 strain) to MEAM1 on the infested leaves of vegetable crops. The current study demonstrated that *H. floridensis* (K22 strain) is a very promising biological control agent for MEAM1 management because the application of *H. floridensis* is highly virulent, e.g., having caused 99.25% mortality of MEAM1 nymphs at 7 dpi. Previous studies have focused on other EPN species against *B. tabaci* in vegetable crops, such as *S. feltiae* (Cuthbertson et al., 2003a, 2003b; Head et al., 2004; Cuthbertson et al., 2007a, 2007b; Ruiz-Platt and Cabello, 2009), *S. carpocapsae* (Cuthbertson et al., 2008; Ruiz-Platt and Cabello, 2009), *H. bacteriophora* (Ruiz-Platt and Cabello, 2009; Meza-García et al., 2014), and *H. indica* (Meza-García et al., 2014). For instance, the applications of *H. bacteriophora* (Guatemala strain), *H. bacteriophora* (Chiapas strain), and *H. indica* (native strain from Sinaloa, Mexico) killed up to 95% of *B. tabaci* nymphs on tomato leaves under laboratory conditions (Meza-García et al., 2014), which was comparable to those obtained from the application of *H. floridensis* (K22 strain) in the current study.

At 3 dpi in the second virulence experiment, the mortality of MEAM1 nymphs caused by the four tested EPN species was low (11.59–30.16%) in the current study. The EPN efficacy against *B. tabaci* was not consistently high in previous studies (Head et al., 2004; Ruiz-Platt and Cabello, 2009). For example, at 2 dpi, the mortality of *B. tabaci* nymphs in pepper caused by EPN (10000 IJs/mL) was reported to be low for *H. bacteriophora* (17.44%), *S. carpocapsae* (35.26%), and *S. feltiae* (18.48%) (Ruiz-Platt and Cabello, 2009). The foliar application of *S. feltiae* (10000 IJs/mL) resulted in 32% and 28% 3dpi mortality of *B. tabaci* nymphs on tomato and cucumber [*Cucumis sativus* L. (Cucurbitales: Cucurbitaceae)] plants, respectively (Head et al., 2004). This low efficacy of EPN species on *B. tabaci* may be due to the short exposure time after treatment applications (2 or 3 days) (Head et al., 2004; Ruiz-Platt and Cabello, 2009) since the EPN efficacy greatly increased at 7 dpi in this study, especially for *H. floridensis* (K22 strain).

We did not detect any host plant effects on the mortality of MEAM1 nymphs between snap bean and tomato at either 3 dpi or 7 dpi. This may be attributed to the optimal environmental conditions (25 ± 2°C, 16 L: 8 D, and 98 ± 2% relative humidity) in our study. Similarly, Cuthbertson et al. (2007b) reported no significant difference on the mortality of *B. tabaci* nymphs caused by *S. feltiae* among tomato, verbena [*Verbena officinalis* L. (Lamiales: Verbenaceae)], cucumber, poinsettia [*Euphorbia pulcherrima* Willd. ex Klotzsch (Malpighiales: Euphorbiaceae)], and chrysanthemum [*Chrysanthemum* × *morifolium* (Ramat.) Hemsl. (Asterales: Asteraceae)] under optimal environmental conditions. However, under sub-optimal environmental conditions, the host plants had significant effect on the virulence of *S. feltiae* against *B. tabaci* due to the leaf surface characteristics (Head et al., 2004; Qiu et al., 2008). For example, under sub-optimal environmental conditions, it was found that mortality of *B. tabaci* nymphs by *S. feltiae* was higher on tomato, cucumber, chrysanthemums, and verbena than on poinsettia (Head et al., 2004). The poor performance of *S. feltiae* on poinsettia may be due to the low hair density and very waxy surfaces, which would reduce humidity along the leaf surface, then reduce nematode survival rate and movement, and consequently cause low mortality of *B. tabaci* nymphs (Head et al., 2004). Thus, further research is needed to determine if our results on host plant effects extend to field conditions.

Toward the development of *H. floridensis* in a biocontrol program for MEAM1, the nematode has several positive attributes as well as challenges. *H. floridensis* has shown high levels of virulence to other arthropod pests such as *Rhipicephalus microplus* Canestrini (Acari: Ixodidae) (Singh et al., 2019). Additionally, *H. floridensis* possesses broad temperature tolerance relative to other EPN species (Shapiro-Ilan et al., 2014) and thus would likely be infective to MEAM1 under hot or cool conditions. However, *H. floridensis*, is not, to our knowledge, currently produced commercially. Thus, production systems would need to be developed and commercial adoption would be required. In the meantime, conceivably another of the nematodes that showed relatively high virulence to MEAM1, and is already in commercial production, might also be tested further for biocontrol potential to MEAM1, such as *H. bacteriophora.*

In conclusion, this study has elucidated that *H. floridensis* (K22 strain) has potential as a biological control agent of MEAM1 on the infested leaves of snap bean and tomato under laboratory conditions. The evaluation of the efficacy of *H. floridensis* (K22 strain) on MEAM1 under more realistic greenhouse conditions requires further investigation. Additionally, more research needs to be explored regarding the effects of adjuvants in reducing the desiccation of *H. floridensis* (K22 strain) and enhancing its persistence. The compatibility of *H. floridensis* (K22 strain) with other natural enemies and/or with chemical insecticides will be also imperative to facilitate the incorporation of *H. floridensis* (K22 strain) into MEAM1 integrated pest management system.
